# Supplementary material for: Null effect of perceived drum pattern complexity on the experience of groove
Source: PLoS One. 2024 Nov 15;19(11):e0311877. doi: 10.1371/journal.pone.0311877 (PMC11567550; doi:10.1371/journal.pone.0311877)
Supplement: S1 Appendix — (PDF) [file pone.0311877.s001.pdf]

APPENDIX

Table 5. Discographic information on the forty drum pattern stimuli

Stimulus	Original Recording (Song Title, Act, Album Title, Year)	Original Drummer	Minutage	Audiofile
1	A Kind Of Magic, Queen, A Kind of Magic, 1986	Roger Taylor	0:26-0:35	01_TayR_2.mp3
2	(Sittin' On) The Dock Of The Bay, Otis Redding, The Dock Of The Bay, 1967	Al Jackson Jr.	0:28-0:39	02_JacA_2.mp3
3	Smells Like Teen Spirit, Nirvana, Nevermind, 1991	Dave Grohl	0:34-0:44	03_GroD_3.mp3
4	Boogie Wonderland, Earth, Wind & Fire, I Am, 1978	Ralph Johnson	0:32-0:41	04_JohR_2.mp3
5	Vultures, John Mayer, Continuum, 2005	Steve Jordan	0:01-0:12	05_JorS_2.mp3
6	Kashmir, Led Zeppelin, Physical Graffiti, 1974	John Bonham	0:18-0:32	06_BonJ_5.mp3
7	Street Of Dreams, Guns N' Roses, Chinese Democracy	Josh Freese	1:13-1:26	07_FreJ_3.mp3
8	Change The World, Eric Clapton, Phenomenon, 1996	John Robinson	2:41-2:53	08_RobJ_3.mp3
9	Let's Dance, David Bowie, Let's Dance, 1982	Omar Hakim	0:08-0:18	09_HakO_3.mp3
10	Space Cowboy, Jamiroquai, The Return Of The Space Cowboy, 1994	Derrick McKenzie	0:07-0:18	10_McKD_2.mp3
11	I Feel For You, Prince, Prince, 1979	Prince	2:44-2:57	11_NelP_5.mp3
12	Virtual Insanity, Jamiroquai, Travelling Without Moving, 1996	Derrick McKenzie	0:09-0:21	12_McKD_3.mp3
13	Bravado, Rush, Roll The Bones, 1991	Neil Peart	0:08-0:21	13_PeaN_4.mp3
14	Let's Go Dancin', Kool & The Gang, As One, 1982	George Brown	1:20-1:35	14_BroG_5.mp3
15	Discipline, Nine Inch Nails, The Slip, 2008	Josh Freese	0:55-1:04	15_FreJ_5.mp3
16	Pass The Peas, The J.B.'s, Food For Thought, 1971	Jabo Starks	0:17-0:28	16_StaJ_4.mp3
17	The Pump, Jeff Beck, There & Back, 1980	Simon Phillips	4:02-4:18	17_PhiS_5.mp3
18	Roxanne, The Police, Outlandos d'Amour, 1978	Stewart Copeland	1:02-1:14	18_CopS_1.mp3
19	Dreamin', Loleatta Holloway, Loleatta, 1976	Earl Young	0:20-0:33	19_YouE_5.mp3
20	Soon I'll Be Loving You Again, Marvin Gaye, I Want You, 1975	James Gadson	1:05-1:17	20_GadJ_1.mp3
21	Summer Madness, Kool & The Gang, Light Of The Worlds, 1974	George Brown	1:52-2:05	21_BroG_4.mp3
22	Listen Up!, The Omar Hakim Experience, We Are One, 2014	Omar Hakim	1:54-2:10	22_HakO_2.mp3
23	Jungle Man, The Meters, Rejuvenation, 1974	Joseph Modeliste	0:00-0:13	23_ModJ_5.mp3
24	Shake Everything You Got, Maceo Parker, Roots & Grooves, 2007	Dennis Chambers	1:52-2:08	24_ChaD_5.mp3
25	Chicken, Maceo Parker, Mo' Roots, 1991	Bill Stewart	0:11-0:24	25_SteB_4.mp3
26	Cissy Strut, The Meters, The Meters, 1968	Joseph Modeliste	0:03-0:16	26_ModJ_3.mp3
27	Far Cry, Rush, Snakes & Arrows, 2006	Neil Peart	0:50-1:03	27_PeaN_2.mp3
28	Alone + Easy Target, Foo Fighters, Foo Fighters, 1994	Dave Grohl	0:22-0:34	28_GroD_5.mp3
29	Soul Man, The Blues Brothers, Briefcase Full Of Blues, 1978	Steve Jordan	0:21-0:31	29_JorS_3.mp3
30	Ain't Nobody, Rufus & Chaka Khan, Stompin' At The Savoy Live, 1982	John Robinson	2:22-2:33	30_RobJ_1.mp3
31	Diggin' On James Brown, Tower Of Power, Soul Vaccination Live, 1998	David Garibaldi	0:08-0:17	31_GarD_5.mp3
32	In The Stone, Earth, Wind & Fire, I Am, 1978	Ralph Johnson	0:53-1:03	32_JohR_3.mp3
33	Southwick, Maceo Parker, Mo' Roots, 1991	Bill Stewart	0:13-0:26	33_SteB_1.mp3
34	You Can Make It If You Try, Sly And The Family Stone, Stand!, 1968	Greg Errico	2:47-2:58	34_ErrG_1.mp3
35	The Dump, Lettuce, Live in Tokyo, 2003	Adam Deitch	5:58-6:10	35_DeiA_3.mp3
36	Killing In The Name Of, Rage Against The Machine, Rage Against The Machine, 1992	Brad Wilk	0:17-0:30	36_WilB_3.mp3
37	Cold Sweat, James Brown, Cold Sweat, 1967	Clyde Stubblefield	0:08-0:18	37_StuC_4.mp3
38	Hyperpower!, Nine Inch Nails, Year Zero, 2006	Josh Freese	0:00-0:16	38_FreJ_4.mp3
39	Rock Steady, Aretha Franklin, Young, Gifted And Black, 1971	Bernard Purdie	2:30-2:44	39_PurB_5.mp3
40	Jelly Belly, Medeski Martin & Wood, Shack-Man, 1996	Billy Martin	0:20-0:31	40_MarB_4.mp3

Note: See also [1], p. 16.

Table 6. Questionnaire items to measure the urge to move (*MOV*, 3 items), temporal regularity (*REG*, 4 items), time-related interest (*INT*, 3 items), pleasure (*PLE*, 3 items), and energetic arousal (*ENE*, 4 items) latent variables. Listeners familiarity (*FAM*) with the stimulus was measured with a single-item scale.

Scale	Item	Stem
MOV	M1	This music evokes the sensation of wanting to move some part of my body.
	M2	This music is good for dancing.
	M3	I cannot sit still while listening to this music.
REG	R1	The rhythm of this music gives a strong sense of regularity.
	R2	The rhythm of this music is steady.
	R3	The rhythm of this music is predictable.
	R4	The rhythm of this music sounds disorganized.
INT	I1	I find the rhythm of this music to be interesting.
	I2	I find the rhythm of this music to be fascinating.
	I3	The rhythm of this music bores me.
PLE	P1	Listening to this music gives me pleasure.
	P2	I like listening to this music.
	P4	Listening to this music is enjoyable.
ENE	E1	This music makes me feel energetic.
	E2	This music makes me feel full of pep.
	E3	This music makes me feel lively.
	E4	This music makes me feel vigorous.
FAM	F1	Have you heard this music in the past?

Notes: The answer categories for the *MOV*, *REG*, *INT*, *PLE*, and *ENE* scales were labeled with: Strongly disagree (0), Disagree (1), Slightly disagree (2), Neither agree nor disagree (3), Slightly agree (4), Agree (5), Strongly agree (6). The answer categories for the *FAM* item were labeled: Definitely not (0), Probably not (1), I do not know (2), Probably yes (3), Definitely yes (4). In the original Experience of Groove Questionnaire, the third item in *PLE* was P3 "This music makes me feel good." This item was discarded, because of its semantic closeness with *ENE*. It was replaced by P4 "Listening to this music is enjoyable." (see also [2], p. 302; [3], p. 54).

Table 7. Measurement model of the latent variables.

Variables	Estimate (γ)	SE	z	p	Standardized Estimate
MOV =~					
M1	1.000				0.892
M2	0.839	0.022	38.521	<.001	0.769
M3	1.061	0.023	46.322	<.001	0.874
REG =~					
R1	1.000				0.901
R2	0.805	0.021	38.171	<.001	0.776
R3	0.934	0.024	39.399	<.001	0.794
R4	0.807	0.026	30.938	<.001	0.669
INT =~					
I1	1.000				0.947
I2	0.944	0.017	54.855	<.001	0.876
I3	0.889	0.020	45.461	<.001	0.797
PLE =~					
P1	1.000				0.920
P2	1.029	0.014	74.559	<.001	0.955
P4	0.992	0.014	71.658	<.001	0.942
ENE =~					
E1	1.000				0.950
E2	0.919	0.013	69.321	<.001	0.904
E3	0.960	0.012	78.721	<.001	0.935
E4	0.936	0.013	72.517	<.001	0.915

Table 8. Covariances between latent mediator variables.

Variables	Estimate	SE	*z*	*p*	Standardized Estimate
REG ~~					
INT	0.127	0.047	2.712	.007	0.073
PLE	0.316	0.044	7.122	<.001	0.192
ENE	0.156	0.048	3.243	.001	0.086
INT ~~					
PLE	1.018	0.055	18.437	<.001	0.543
ENE	0.999	0.059	16.920	<.001	0.482
PLE ~~					
ENE	0.951	0.056	17.125	<.001	0.485

Table 9. Error variances (or uniquenesses) of the indicator and endogenous variables.

Variable	Estimate	SE	*z*	*p*	Standardized Estimate
M1	0.564	0.035	16.031	<.001	0.204
M2	1.069	0.043	24.658	<.001	0.409
M3	0.764	0.042	17.994	<.001	0.236
R1	0.438	0.032	13.834	<.001	0.188
R2	0.812	0.034	23.662	<.001	0.397
R3	0.971	0.042	22.852	<.001	0.370
R4	1.528	0.058	26.472	<.001	0.553
I1	0.274	0.026	10.459	<.001	0.103
I2	0.647	0.031	20.531	<.001	0.233
I3	1.082	0.043	25.299	<.001	0.364
P1	0.367	0.017	22.181	<.001	0.153
P2	0.210	0.013	15.818	<.001	0.089
P4	0.254	0.014	18.699	<.001	0.113
E1	0.269	0.014	18.611	<.001	0.098
E2	0.467	0.019	24.220	<.001	0.183
E3	0.329	0.015	21.214	<.001	0.126
E4	0.419	0.018	23.380	<.001	0.163
MOV	1.140	0.055	20.791	<.001	0.518
REG	1.533	0.069	22.292	<.001	0.807
INT	1.980	0.079	25.116	<.001	0.830
PLE	1.772	0.071	24.818	<.001	0.873
ENE	2.166	0.082	26.288	<.001	0.879

References

[1] Senn O, Hoesl F, Jerjen R, Bechtold TA, Kilchenmann L, Rose D, et al. A Stimulus Set of 40 Popular Music Drum Patterns with Perceived Complexity Measures. Music & Science. 2023 Jan 1;6:20592043231202576.

[2] Senn O, Bechtold T, Hoesl F, Jerjen R, Kilchenmann L, Rose D, et al. An SEM approach to validating the psychological model of musical groove. Journal of Experimental Psychology: Human Perception and Performance. 2023 Mar;49(3):290–305.

[3] Senn O, Bechtold T, Rose D, Câmara GS, Düvel N, Jerjen R, et al. Experience of Groove Questionnaire: Instrument Development and Initial Validation. Music Perception. 2020 Sep 9;38(1):46–65.
