# Supplementary material for: Null effect of perceived drum pattern complexity on the experience of groove
Source: PLoS One. 2024 Nov 15;19(11):e0311877. doi: 10.1371/journal.pone.0311877 (PMC11567550; doi:10.1371/journal.pone.0311877)
Supplement: S1 Text — (PDF) [file pone.0311877.s002.pdf]

RE-ANALYSIS OF THE WITEK ET AL. (2014) DATA

Olivier Senn and Maria Witek

Introduction

This document provides a re-analysis of the data collected by Witek and colleagues for their 2014 study “Syncopation, Body-Movement and Pleasure in Groove Music” [1]. The goal of the re-analysis is to better interpret the results of the new study (presented in the main article) by finding a common ground between the stimuli and the results of the two studies.

The stimuli of [1] consist of 50 drum patterns with bass drum, snare drum, and hi-hat voice. All patterns are in 4/4 common time and played at a tempo of 120 bpm. The loudness of each instrument is fixed, and note onset times are metronomically precise (there are no microtemporal deviations). In all patterns, the hi-hat voice presents a regular pulse of eighth notes, while the bass drum and snare drum provide a variety of rhythms. Each pattern is associated with a numeric value on the *Index of Syncopation* (a heuristic measure of rhythmic complexity for each drum pattern, based on syncopation in the snare drum and bass drum voices).

Of the 50 drum patterns used in Witek et al. [1], 36 were modelled after drum patterns found in Western popular music (= drummer-composed), with the snare drum and bass drum voices reproducing the rhythm of these instruments in the original recording; some drum patterns were provided by the sequencer software GarageBand. The remaining 14 drum patterns used the same instrumentation, but the bass drum and snare drum voices were created by the researchers (= experimenter-composed) with the goal to obtain very low or very high

values on the *Index of Syncopation*. Due to the fact that the hi-hat played a regular eighth-note pulse in all stimuli, it did not add syncopation to any of the drum patterns.

The stimuli sets of the new study (presented in the main article) and of Witek et al. [1] show great similarities: all stimuli present drum patterns with an instrumentation of bass drum, snare drum and cymbals. The major differences are that the new stimuli are exclusively based on drum patterns from the Western popular music repertoire (drummer-composed), and they show an increased detail in their replication of the originally recorded patterns (cymbal patterns, dynamics, tempo, microtiming), in comparison with the stimuli in [1].

In a first analysis, we will fit a quadratic regression model to the *urge to move* ratings of the entire Witek et al. [1] dataset in order to replicate their published result. In a second analysis, a quadratic regression model will exclusively be fitted to the *urge to move* ratings of the drummer-composed patterns. This second analysis appears to be most appropriate for a comparison between the two studies.

Analysis 1: All stimuli

In the first analysis, we fit a quadratic regression model to the entire Witek et al. [1] data set with $N = 3300$ ratings from $n = 66$ participants on all 50 stimuli, using the *syncopation* (as measured by the *Index of Syncopation*) and *squared syncopation* values as predictors for the *urge to move* ratings. **Table 1** shows the coefficients of the best-fitting quadratic model. All coefficients are significantly different from zero. The coefficient of *squared syncopation* is negative, indicating an inverted-U curve.

Table 1. Quadratic models predicting *urge to move* ratings from *Syncopation* and *Syncopation*^{2} for the entire Witek et al. [1] data set (drummer- and experimenter-composed patterns).

Source	Estimate	SE	t	*p*
Intercept	2.30281	0.05876	39.19	<.001
Syncopation	0.04553	0.00354	12.88	<.001
Syncopation^2	–0.00066	0.00004	–14.87	<.001

Notes: SE: standard error; t: t-statistic; p: significance probability.

Figure 1 shows a scatterplot of the $N = 3300$ *urge to move* ratings (y-axis) against the patterns' *index of syncopation* (x-axis) as small semi-transparent dots in the background, where the colors identify responses to drummer-composed (blue) or experimenter-composed (turquoise) drum patterns. Stimuli means are given as white dots with error bars that represent the standard error of the mean. The regression parabola (white curve) corresponding to the model of **Table 1** has an inverted-U shape. It accounts for a substantial amount of the variance in the ratings ($F_{(2,3297)} = 126.0$, $p < .001$, $R^2 = .07$), and it has its apex at a medium value of the *index of syncopation* (34.2).

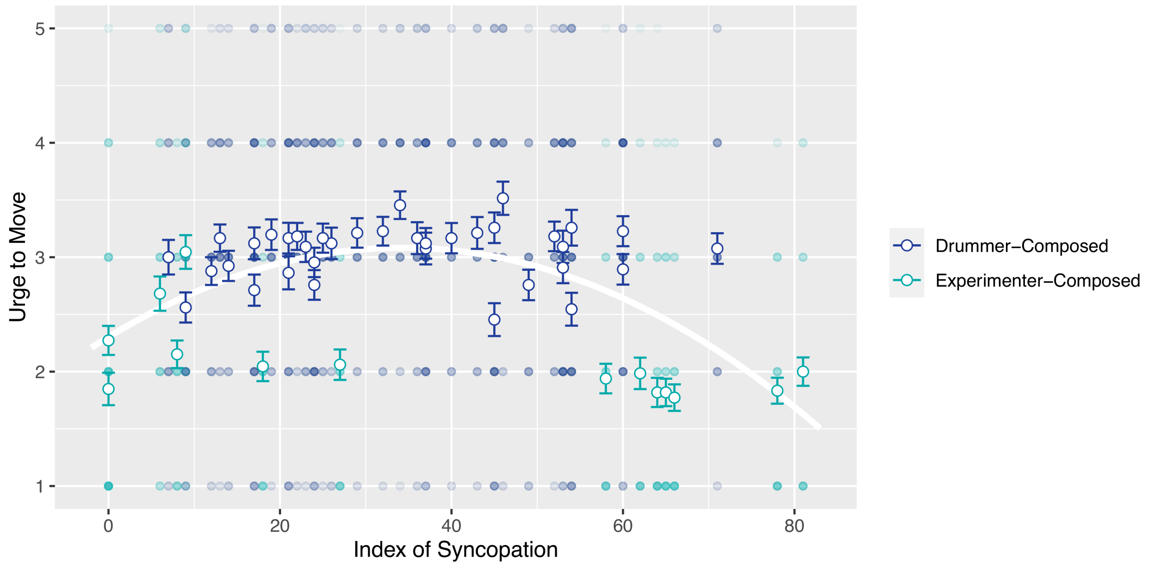

Figure 1. All drum patterns. Background: Single *urge to move* (y-axis) ratings are shown as small semi-transparent points against the *index of syncopation* (x-axis). Foreground: Mean *urge to move* ratings are given for each stimulus (white dots with error bars that represent the standard error of the mean) and the regression parabola of the best-fitting quadratic model (white curve). Data relating to drummer-composed patterns are blue, data relating to experimenter-composed patterns are turquoise.

Analysis 2: Drummer-composed patterns

In the second analysis, we focus on a subset of the data ($N = 2376$) relating to the 36 drummer-composed patterns (the blue markings in **Figure 1**). **Table 2** shows the coefficients of the best-fitting quadratic model that regresses the *urge to move* ratings on the patterns' *syncopation* and *squared syncopation* values.

Table 2. Quadratic models predicting *urge to move* ratings from *Syncopation* and *Syncopation*^{2} for the subset of the Witek et al. (2014) data that relates to drummer-composed patterns.

Source	Estimate	SE	t	*p*
Intercept	2.75489	0.10195	27.022	<.001
Syncopation	0.01824	0.00629	2.900	.004
Syncopation^2	–0.00023	0.00008	–2.716	.007

Notes: SE: standard error; t: t-statistic; p: significance probability.

The best-fitting regression parabola has an inverted-U shape (**Figure 2**) with its apex at a medium syncopation level of 39.6. The model explains a significant amount of the variance in the *urge to move* ratings ($F_{(2,2373)} = 4.338, p = .013$), yet the effect of syncopation on the urge to move is very small ($R^2 = .003$).

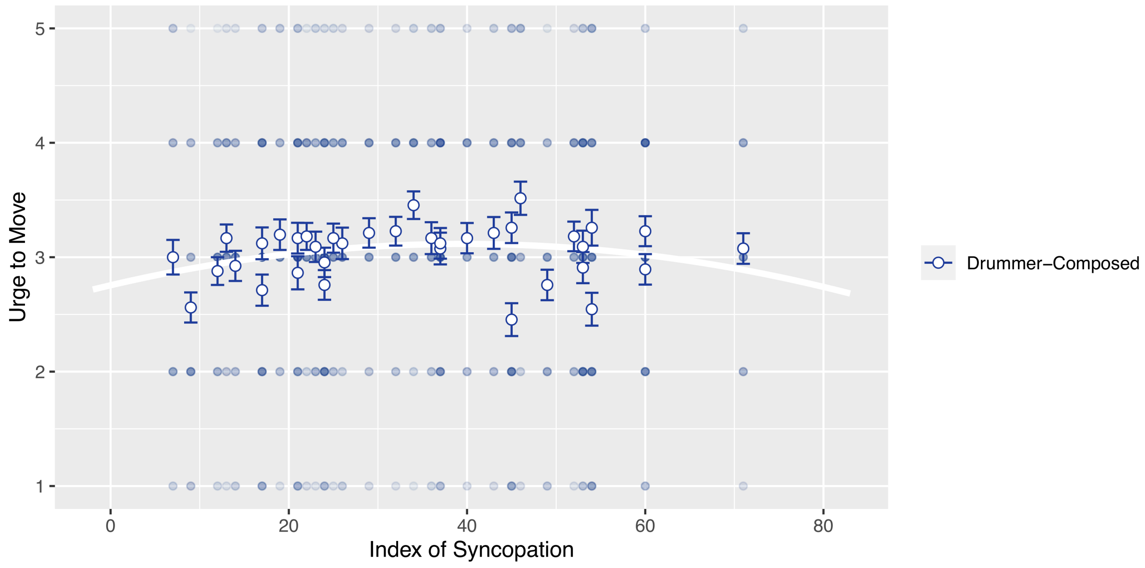

Figure 2. Drummer-composed patterns only. Background: Single *urge to move* (y-axis) ratings are shown as small semi-transparent points against the *index of syncopation* (x-axis). Foreground: Mean *urge to move* ratings are given for for each stimulus (white dots with error bars that represent the standard error of the mean) and the regression parabola of the best-fitting quadratic model (white curve).

Conclusions

The two analyses confirm the result of the Witek et al. study [1] that the urge to move in response to popular drum patterns is an inverted-U function of the index of syncopation in the bass drum and snare drum patterns. The second analysis adds the nuance that the inverted-U relationship is very weak if we only include drum patterns that have their origin in a practical popular music context such as commercial recordings or applications such as GarageBand (drummer-composed patterns).

Reference

[1] Witek MAG, Clarke EF, Wallentin M, Kringelbach ML, Vuust P. Syncopation, Body-Movement and Pleasure in Groove Music. PLoS One. 2014 Apr 16;9(4):1–12.
