# Supplementary material for: Null effect of perceived drum pattern complexity on the experience of groove
Source: PLoS One. 2024 Nov 15;19(11):e0311877. doi: 10.1371/journal.pone.0311877 (PMC11567550; doi:10.1371/journal.pone.0311877)
Supplement: Attachment — Submitted filename: Rebuttal_Letter_240830.docx [file pone.0311877.s003.docx]

**Rebuttal Letter**

Dear Reviewers, dear PLOS One Academic Editor

Many thanks for engaging with our study so closely and for offering detailled, thorough, and constructive commentary on so many aspects of the paper. This is peer-review at its best: we highly appreciate the effort that you put in the reviews, and we don’t take it for granted.

We respond in kind by re-submitting the paper with many major and minor revisions; and we hope the new version addresses your concerns in a satisfactory way. You’ll find our direct response to your critique and recommendations in this rebuttal letter below, where line numbers (L…) point to changes in the manuscript.

The most substantial changes can be found in the Discussion section: large parts were rewritten from scratch in order offer a more in-depth engagement with our own results and to better connect them to (or contrast them with) other findings in the field.

We appreciate that you take the time to engage with our study yet another time, and we are looking forward to your comments and further criticism.

Kind regards

Olivier Senn & Co-authors

**Reviewer 1**

*The study is relevant and interesting. It is very well crafted and presented.*

Thank you for your appreciation of the study in general and for your very motivating comments – we appreciate it!

*“Why was the inverted-U hypothesis not confirmed in this study?” Unfortunately, the biggest portion of this section does not answer this question but rather discusses why earlier studies might have found it in contrast to the present one. […] It reads almost as if they wanted to explain away their main finding rather than discuss it.*

Upon re-reading the Discussion section of our first submission we agree with you that this section was too superficial. It lacked a coherent argument that connects our study with past research, and we obviously shied away from drawing inconvenient conclusions about the inverted-U hypothesis.

To address your critique, we rewrote the entire first part of the Discussion in L531-655. The new version takes a closer look at the stimulus set of our own study and of other studies that investigate the inverted-U hypothesis. We argue that the complexity of the stimuli in most of the replications is confounded with the different levels of ecological validity among the stimuli: Medium-complexity stimuli tend to be based on idiomatic musical patterns created by musicians, while, in many studies, most of the low and high complexity stimuli are based on potentially un-idiomatic patterns created by researchers. We suggest that the inverted-U relationship between complexity and the urge to move found in many studies may be an effect of using idiomatic and un-idiomatic stimuli in the same experiments. We hypothesize that – in accordance with our results – there may not be a systematic relationship between the complexity of popular music drum patterns and their capacity of motivating listeners to move, as long as we only consider idiomatic patterns with high ecological validity. With your permission, we gladly use your observations on the existence of simple and complex dance music and the role of familiarity to support this point (L643-647).

Let us know to what extent the rewrite solves the problems that you identified in the first part of the Discussion section. The section now runs a bit long and potentially presents too much detail. This is not a serious problem, because PLOS does not impose an upper word count limit, yet conciseness is a valued property nevertheless. If you see parts that can safely be shortened, we are grateful for your advice.

*The authors mention that a possible limitation of the study is that the stimuli were modeled after performances by highly competent musicians. In the same vein, they stated earlier in the discussion that “Great drummers can play a simple pattern in such a way that it is still interesting to the listener, and they ca [sic] present a complex pattern such that listeners still feel the regularity.” I am skeptical about this representing a limitation.*

The reviewer observes that this point was mentioned twice, once in the Discussion and another time in the Limitations sections. We omitted the first mention of this point in the Discussion and made it more concise in the Limitations (L718-728). In general, we concur with the reviewer that the performance skill of the performer is not likely to affect the results, since the reproduction only captures a few aspects of the individual nuances of the actual performance. We removed this point from the text, since we cannot offer a valid assessment of how close the reproductions are to the original performances (other than relating that we tried to make them as good as possible, L177-194). Nevertheless, we still think there might have been some kind of selection bias: choosing only renowned drummers and famous/iconic patterns may have an effect onthe relationship between complexity and the urge to move. We tried to clarify this thought in L718-L728.

*The authors then mention as a limitation that the stimuli cover a range of complexity that is limited to the range that is typical of the represented genres. Again, I do not think this is a limitation of the study but rather adds to its ecological validity.*

Agreed. We left this as a limitation (L718-L728), but focused more on the aspect that we used mainstream popular music and did not consider more experimental genres.

*Alongside the above limitations relating to the details of the auditory stimuli used in the present study, it might also be worth going one level of abstraction higher and reflecting on the concept of psychological groove research a bit more generally. The following limitations concern the present study and the field at large.*

Reductionism is a generalized problem in groove research: stimuli in experiments are usually thinned, shortened and simplified shadows of what music is in the real world. The repertoire is often conventiently restrained to specific genres, and the experimental listening situations often have little in common with the ways people usually experience music (with a few notable exceptions in which groove research managed to use real music in a real listening situation, such as Swarbrick et al, 2019; Cameron et al., 2022). Reductionism not only affects the stimuli (drum set vs. full band) and musical styles (rock, pop, funk vs. everything else), but also the perception modalities (auditory only vs. multimodal) and the listening situation (filling a questionnaire at a computer vs. moving freely to music in a real-world situation). We took the liberty to pack all your points about the problems of reductionism into one big limitation (L746-761). We are aware of these problems, and it does not hurt to state them explicitly.

The groove model framework and its implementation with scales and SEM etc. lead to a fairly complicated data collection procedure, which, unfortunately, is not improving the listening experience for the participants (stimuli in small chunks, lots of repetitions, lots of ratings). Thus the model adds to the problem that the online survey situation is not a natural listening situation – we acknowledge this in L739-745. We just hope that the perks of the model (simultaneous testing of a constellation of direct and indirect effects) at least allows to better contextualise the groove experience in the long run and improve our understanding of the phenomenon.

*Smaller points:*

*Danceability*: We introduced danceable as a short form of “which motivates dancing”. But we agree that this might be a bit confusing, so we replaced the expression with longer, but hopefully clearer formulations (L17, L18).

L231: “the music wit” → “musical stimulus with”. Corrected in L267.

L233: “to what extent feel” → “to what extent listeners feel”. Corrected in L273.

L477: “they ca represent” → “they can represent” Sentence removed during rewrite.

L526: “predicotr” → “predictor”. Corrected in L672.

**Reviewer 2**

Many thanks for your excellent suggestions. We have made the following changes to the manuscript in order to address your comments:

*1) The authors did not test (or show) the relationship between syncopation and MOV level for their stimuli.*

Thank you for highlighting the *Syncopation* → *MOV* relationship. We had a short passage about this aspect in the originally submitted manuscript, but we gladly present it a bit more prominently. We have added more detail in the text (L408-L429) plus a new figure (Figure 3), which shows that this relationship turns out to be “U”, but not “inverted-U”.

*Additionally, I wonder if the range of syncopation in the dataset is too narrow to reveal a clear inverse U-shaped curve.*

We added information about the range on the Index of Syncopation in L406-409. Note that we had to change this metric to *Syncopation per beat* in order to make the syncopation values of the two studies compatible with each other (Witek et al.’s patterns have a length of 2 bars, whereas the patterns in the current new study have a length of 4 bars), so simply summing up syncopation values would be misleading .

It turns out that the range of the Syncopation per beat values in Witek et al. (2014) of [0.0, 10.1] (L414) are indeed 30% larger than the range in the new study [0.5, 8.3] (L412). If we only consider drummer-composed patterns, then the range of syncopation values of Witek et al. is [0.875, 8.875] and thus quite similar to the range in the new study.

Note that we reframed the argument in the first part of the Discussion section (L531-655) in order to better explain our take on the differences between the replication studies that confirmed the inverted-U and our new study. Our main point is that previous replications use idiomatic musical patterns in the mid-complexity range, and newly composed, potentially unidiomatic patterns in the low- and high-complexity domains. This also addresses the complexity range, since the un-idiomatic patterns are situated at the extremes of the syncopation dimension.

*2. It would greatly enhance the readability of the article to better explain this measure and how it diverges from the level of syncopation in the methods section.*

We agree completely on this point: we added a minimal set of explanations on the complexity measure in L195-237, and directing the reader to Senn et al. (2023a) for more more detailled information. Let us know if this is sufficient or whether more detail should be given.

*3. Perceived complexity seems to be a subjective measure of musical stimuli. Could the absence of an inverse U-shaped relationship between this metric and MOV be due to potential covariations?*

We are not entirely sure whether we understand your question correctly. We agree that the Syncopation Index is an objective measure (i.e. it only depends on the properties of the drum pattern itself), and perceived complexity is a subjective measure (i.e. it depends on the perception and judgement of people hearing a pattern). The two measures are positively correlated with each other in the case of our stimuli (r=0.7), but they are not identical. Our data did not confirm the inverted-U-hypothesis in either case, regardless of whether we regressed *MOV* on the Index of Syncopation or on Perceived Complexity.

What do you mean by covariations? Do you think of any specific co-variate that we should consider and that, if included, might lead to a different result? If so, please elaborate which co-variates might be interesting to test.

*Minor Revisions*

1. “the music wit” → “the music with”: corrected (L267).

2. “predicotr” → “predictor”: corrected (L672).

3. “SEM”: thank you for catching this, we have added the complete formulation to the first appearance of the acronym (L123).

**Reviewer 3**

*First of all, I would like to thank the authors for their excellent paper, which I have read with great interest and pleasure.*

Many thanks for your kind words and constructive comments!

*This result is very similar to that reported by Sioros et al 2022, where the original syncopated patterns of commercial songs were rated higher than algorithmically syncopated patterns of the same songs.*

Thank you for (re-)directing our attention to the Sioros et al. (2022) study. While preparing our experiment, we were aware of this study, but we did not yet understand how much its design and results help with the interpretation of our results. We have rewritten large portions of the Discussion session (L531-655) in order to give a clearer interpretation of the results (also in response of the remarks of other reviewers). In the course of this rewrite, we also added a paragraph (L605-636) on the Sioros et al. (2022) study: this allows us to put the entire discussion on idiomatic vs. un-idiomatic patterns into perspective.

*[…] I wonder if some analysis regarding cross-rhythms and pickups could also be done in the Witek et al. (2014) dataset.*

Indeed, it would be very interesting to carry out this kind of analysis. This might start with the statistical analysis of a larger corpus (e.g. the drum patterns published by Hosken et al., 2021) that would offer a great basis for the description of syntax. Concrete musical patterns from stimuli set such as Witek et al., (2014) could then be discussed with respect to the syntax. Given that this would be a major enterprise and that the current paper is already very long, we refrain from doing any type of analysis in this direction. However, we suggest it should be done in the future (L632-636).

*2) I find that lines 8-10 of the abstract are not very clear as to what the novel contribution of this paper is.*

We agree, this was not yet to the point. We tried to formulate this more clearly (L6-14). What do you think?

*3) A reference to a definition of syncopation may be needed in lines 80-82.*

We added references and adapted our definition of syncopation in order to better link the references and PCRI (L74-80).

*4) But doesn't this mean that other features of music are actually more important than complexity for our urge to move?*

We agree entirely: there certainly are other aspects that are important for the groove experience, besides complexity (which potentially is not very relevant at all, see L640-643). In the mentioned passage of the first submissien we intended to point to performative aspects that are likely to be relevant. Note that this passage was removed in the course of the rewrite, but some ideas can be found in the Limitations section (L718-728).

*5) Syncopation is not linearly related to complexity, even though complexity and syncopation have sometimes been used almost interchangeably in the literature. […] I do not disagree with the specific lines in the abstract, but perhaps this should be further clarified in the text […]*

We have added a critique of syncopation as a measure of complexity already in the abstract (L8-10). Mentioning that syncopation is not a perfect measure of complexity added to our motivation of carrying out yet another replication with new stimuli and a perceptual measure of complexity. We also reformulated the corresponding passage in the Introduction to better differentiate syncopation and complexity (L65-L70).

*6) line 510-512: This is an excellent result! I would even remove the word "every-day". The experience of groove is genre specific, even if there may be some universal principles behind it.*

Thank you! We slightly reformulated this point in the course of the rewrite of the Discussion. “Every-day” came from the idea of contrasting the lab situation with listening situations in the real world that should be the focus of our investigations. We tried to make this contrast a bit stronger (L746-761).

“they ca represent” → “they can represent”: Thank you for catching this, the sentence was removed in the course of rewriting the Discussion section.

**Reviewer 4**

*Firstly, I like this study very much. The study has clear hypotheses and appropriate methods of analyses. Additionally, the results are interesting and advance our understanding of the relationship between the groove experience and rhythmic complexity.*

Many thanks for your kind assessment of the study as a whole, and especially for your constructive and helpful comments!

Note, that in response to reviewers’ comments we have substantially changed the Discussion section to give a more comprehensive interpretation of the results (L531-L655), which was too superficial in the first submission.

*1) Therefore, my suggestion for interpreting the results is that there is an inverted U-shaped relationship when examining the general relationship between groove experience and rhythmic complexity (as shown by Witek et al., 2014). However, when focusing on idiomatic rhythms (or music), this relationship does not appear.*

Thank you for this interesting thought, which could present a salomonic way to solve the discrepancies between the current study and previous replications. Do we understand you correctly: you suggest that the inverted-U relationship between complexity and the urge to move may be generalized across all musics (and all listeners) of the world, but it does not appear when only music from one single musical idiom is considered?

In our study we only can provide evidence for the second part, since we only considered one type of stimuli (drum patterns from Western popular music). To our knowledge, research has not yet provided evidence for the first part of the hypothesis: none of the previous studies have used stimuli sets that are not to a certain degree homogenous with respect to stylistic aspects.

The practical challenges of designing a study that provides valid evidence for the generalized inverted-U hypothesis across many genres would be formidable: how to select a stimuli set that represents music in such a generalized way? We can easily think of stimulus sets that result in an inverted-U relationship (low complexity: meditation music / medium complexity: funk / high complexity: Xenakis), but similarly easily we could think of sets that trigger the inverse reaction (low: eurodisco / medium: symphony slow movements / high: salsa). The problems of selection bias appear to be quite insurmountable. How would researchers deal with the infinity of potentially confounding variables (musical properties, the modi in which the music is usually listened to, listeners’ taste, familiarity etc.). We think the generalized inverted-U hypothesis is an interesting idea, but for the time being we do not see a method to succesfully investigate it.

*2) You stated, "the study found a psychological mechanism that potentially explains how the inverted-U shaped hypothesis works, but it could not confirm the hypothesis itself." Why do you think this happened?*

Upon re-reading the passage, we were not happy with the way we formulated this. The sentence made readers think that the inverted-U was an inevitable consequence of H2 and H3 being true, but this is simply not the case. We now chose a more neutral/toned-down formulation to report this result (L787-L794).

*3) I have a concern that the perceived complexity used in this study does not come from the participants of this study (as you mentioned in the limitations). Is there any reason you did not collect the perceived complexity data from the participants this time? Are the results of the previous study regarding perceived complexity rigorous?*

Thank you for this remark. Other reviewers also commented that the perceived complexity measure was not sufficiently introduced in the paper. Accordingly, we have added two paragraphs to the stimuli section (L195-237) that better explain these values, how they were calculated, and what they mean. Let us know, if this improves the situation.

The complexity and groove measurements could not be carried out in the same experiment, because both were full-size listening experiments. In our opinion, the complexity measurement was indeed very rigourous: It used a pairwise comparison approach in which participants listened to 20 pairs of stimuli and chose which of the two sounded more complex. Participants tend to solve this kind of ranking tasks with considerable ease. The participants don’t need to be “fair” across trials as in Likert rating: every trial provides the full information that is necessary to carry out the task. The pairwise comparison data was then analysed using the Bradley-Terry probability model, which provided the perceived complexity estimates. These estimates allow to calculate a probability that one stimulus wins a pairwise contest over another stimulus in a pairwise comparison contest, when judged by a member of the surveyed population. So, as long as it is plausible that the two studies’ participant groups were sampled from (more or less) the same population and as long we have the probabilistic interpretation of the complexity measures in mind, the estimates should be accurate across samples.

Minor comments

*1) It is better to cite a reference such as Vuust et al. (2022).*

We referenced Vuust et al. (2022) earlier in the text, when the PCRI theory is discussed (L73), but left the reference to Stupacher et al. (2022) at L88, because the “sweet spot” quote is verbatim from this publication.

*2) Do you have a citation for the following statement?
"Synchronized body movement (resp. dancing) allows a listener to process rhythmic complexity more easily"*

Good point: For the statement in this general form we do not have any hard evidence. So, we toned the statement down and referenced a fitting result from the multi-sensory perception literature (L102-105).

*3) line 129 "a series psychometric scales" → "a series of psychometric scales"?*

Thanks for catching this – corrected (L129)

*4) line 231 "wit" → "with"*

Corrected (L267)

*5) line 270 Does "s" mean "SD" or "SE"?*

The value is a standard deviation – we corrected this to SD (L310, and also L317)

*6) line 300 Why did you always present the MOV & FAM block first?*

MOV is the main response variable of the study. By presenting it in the first we could make sure that participant were still fresh, when they rated the stimuli on MOV. We wanted to measure FAM in the first block as well. This item was: “Have you heard this music in the past?” with answer categories “Definitely not,” “Probably not,” “I do

not know,” “Probably yes,” and “Defnitely yes”). Consequently the question can only be asked in the first block, not later when participants had heard ir before and rated it on a different variable. Additional explanations are given in L339-342.

*7) line 362 What is the range of the calculated syncopation in this study? The readers (including myself) would likely be interested in the difference of the range between this study and Witek et al. (2014).*

Thank you, we added this information (L408-418), yet we had to calculate Sycopation per beat values for the two studies in order to make the measures compatible (Witek et al., 2014, used 2-bar patterns, whereas we used 4-bar patterns.) The bottom line is: Witek et al. (2014) used a substantially greater range of syncopation compared to our stimulus set. However, the ranges are very similar when the experimenter-composed patterns are removed.

*8) line 398 Is it possible to investigate non-linear relationship (e.g., quadratic relationship) using the SEM approach? You don't have to try this for this study, but I was just curious if the time-related interest linearly increases as the complexity becomes higher. There might be an inverted-U relationship between the two.*

Yes, non-linear relationships can be modelled in SEMs (see link below). However, I (OS) haven’t tried that yet. I would also be interested to know whether two effects are necessarily additive in SEMs, or whether there are ways to model multiplicative effects. This would be necessary to express the Prediction Errors x Prediction Certainty = Certainty-Weighted Prediction Errors of PCRI.

<https://www.tandfonline.com/doi/full/10.1080/10705511.2020.1857255>

*9) It is plausible to find a null effect of complexity if we assume that the complexities of the rhythms used in this study fall within the optimal range.*

It is possible that our stimulus set hits the central, quasi horizontal part of the inverted-U curve, assuming that excellent drummers are capable of presenting highly effective patterns regardless of the complexity (see the limitation in L718-728). It is unclear what would happen if these patterns were played by mediocre drummers. Would the inverted-U appear, because they are not able to play the complex patterns well and because they perform simple patterns in a lame way? But the medium complexity patterns obtain higher ratings? (Why would that be the case?) Maybe the ratings would just drop across the whole complexity range? So far we do not have data to investigate this question.

*10) line 517 I think that if you want to cite them, you should wait for them to be published. Please check the PLOS ONE guidelines once again.*

We have removed these references for the now. We hope that preprints will be available when the peer review process of the current study comes to an end, so we can put them back in.

*11) line 526 "predicotr" → "predictor"*

This was corrected (L672)

*12) line 542*

*Please also check if "Kawase et al., in press" can be cited.*

Thank you, we will check this as well (L688): the Kawase paper had a first round of reviews, and it could go through before the current one.
